# Supplementary material for: Denoising and iterative phase recovery reveal low-occupancy populations in protein crystals
Source: Commun Biol. 2025 Nov 24;8:1649. doi: 10.1038/s42003-025-09031-6 (PMC12644873; doi:10.1038/s42003-025-09031-6)
Supplement: Supplementary file 2 — Supplemental Information [file 42003_2025_9031_MOESM2_ESM.pdf]

Supplementary Information

Denoising and Iterative Phase Recovery Reveal Low-Occupancy Populations in Protein Crystals

Alisia Fadini[1*,+], Virginia Apostolopoulou[2,3], Thomas J. Lane[2,3*], Jasper J. van Thor[1*]

[1]Department of Life Sciences, Faculty of Natural Sciences, Imperial College London, London SW7 2AZ, United Kingdom

[2]Center for Free-Electron Laser Science CFEL, Deutsches Elektronen-Synchrotron DESY, Notkestr. 85, 22607 Hamburg, Germany

[3]The Hamburg Centre for Ultrafast Imaging, Luruper Chaussee 149, 22761 Hamburg, Germany

[+]Current address: Department of Systems Biology, Columbia University, 622 West 168th St., New York, NY 10032, USA

[*]Correspondence

Supplementary Notes

S1. Validation of the Choice of Regularization Parameter for Total Variation Denoising

As introduced in the main text, the choice of the λ regularization parameter is critical in total variation (TV) denoising; it determines the level of denoising at the expense of fidelity to the original data, as formulated by the original expression for the TV minimization problem by Rudin, Osher, and Fatemi [1] and implemented by Chambolle [2]:

$$\min_u \sum_{i=0}^{N-1} \left(|\nabla u_i| + \frac{(s_i - u_i)^2}{2\lambda} \right) \tag{1}$$

where the objective is to recover the signal u, characterized by a lower total variation (defined as $\sum_{i=0}^{N-1} |\nabla u_i|$) than the original signal s, while ensuring u closely resembles s in an L^2 sense. For λ close to zero, the output signal is almost unchanged from the original. As λ increases, the term minimizing total variation dominates, leading to increased smoothing and decreased fidelity to the input data.

To determine an optimal regularization value for a DED map, we propose selecting the value of λ that maximizes the negentropy of the voxel value array. We validate this approach by calculating a synthetic *trans*-to-*cis* difference density map (Figure S3(a)). To introduce noise, we add random values to the *trans* and *cis* structure factors, drawn from a Gaussian distribution with a mean of 0 and a standard deviation equal to 10% the standard deviation of the structure factor magnitudes, to resemble experimental maps. The real space map displays the signal as well as the expected added noise (Figure S3(b)).

Figure S3(c) presents the negentropy values obtained by denoising the map with a range of regularization weights. Notably, the maximum negentropy corresponds closely to the value of λ that also maximizes the real-space Pearson correlation coefficient between the denoised map and the ground truth synthetic map density (ρ_{true}).

S2. Validation of Iterative Total Variation Denoising with a Synthetic Noisy Map

In addition to the experimental data presented in the main text, we also test the iterative-TV density modification technique (it-TV) on the synthetic noisy map described in the previous section (Figure S3(b)). it-TV produces a map that preserves the expected positive and negative densities while significantly reducing noise (Figure S3(d)). The diagram in Figure S3(d) compares the noise-free ground truth map (ρ_{true}) with the denoised map at each iteration (ρ_{itTV}). As ρ_{itTV} iteratively improves, it more closely approximates ρ_{true}, with its negentropy converging to a maximum.

This alignment between negentropy optimization and the maximization of the Pearson correlation coefficient with ρ_{true} in synthetic noisy data for both TV and it-TV treatments further supports the use of negentropy as an effective quality statistic.

Supplementary Figures

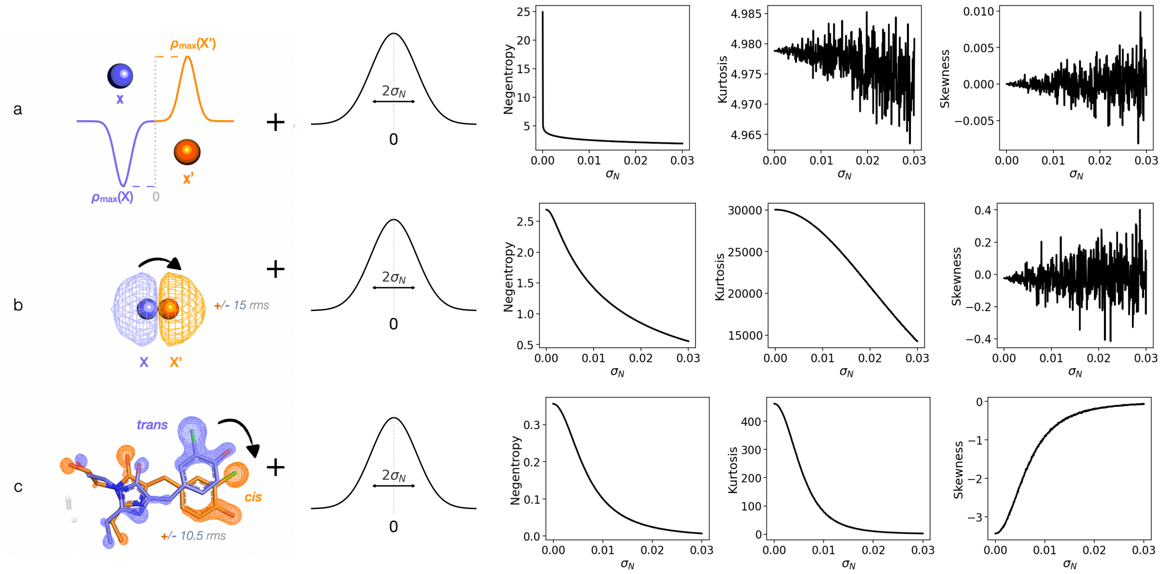

Figure S1. Quantitative indicators of Gaussianity in simulated difference electron density (DED) maps as a function of noise fraction. We add random noise to the real space density maps of the simulated examples from the main Figure 1. Noise is simulated as a standard normal distribution (mean 0, variance σ_N). We compute negentropy, kurtosis, and skewness for the distribution of map voxel values maps at increasing levels of σ_N. We find that find that negentropy decreases monotonically with the addition of noise and is the most robust in its behavior when compared to skewness and kurtosis across the three model examples. On the basis of this test, we proceed with the proposal that negentropy could be a useful metric to evaluate the signal-to-noise ratio in difference density maps.

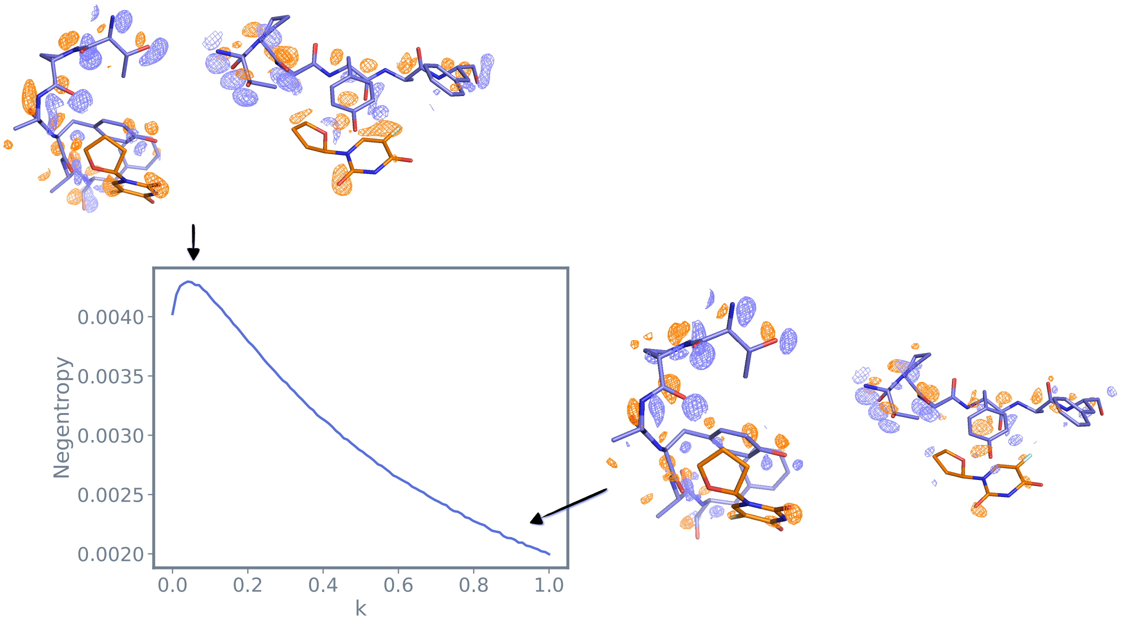

Figure S2. Negentropy can be used for parameter determination in k-weighting of difference structure factors. The choice of the *k* parameter for outlier rejection in k-weighting [3,4] has so far been determined by user visual inspection. We show here that negentropy maximization can be used to choose the *k* value for the M^{pro}-tegafur data (PDB ID 7AWR) .

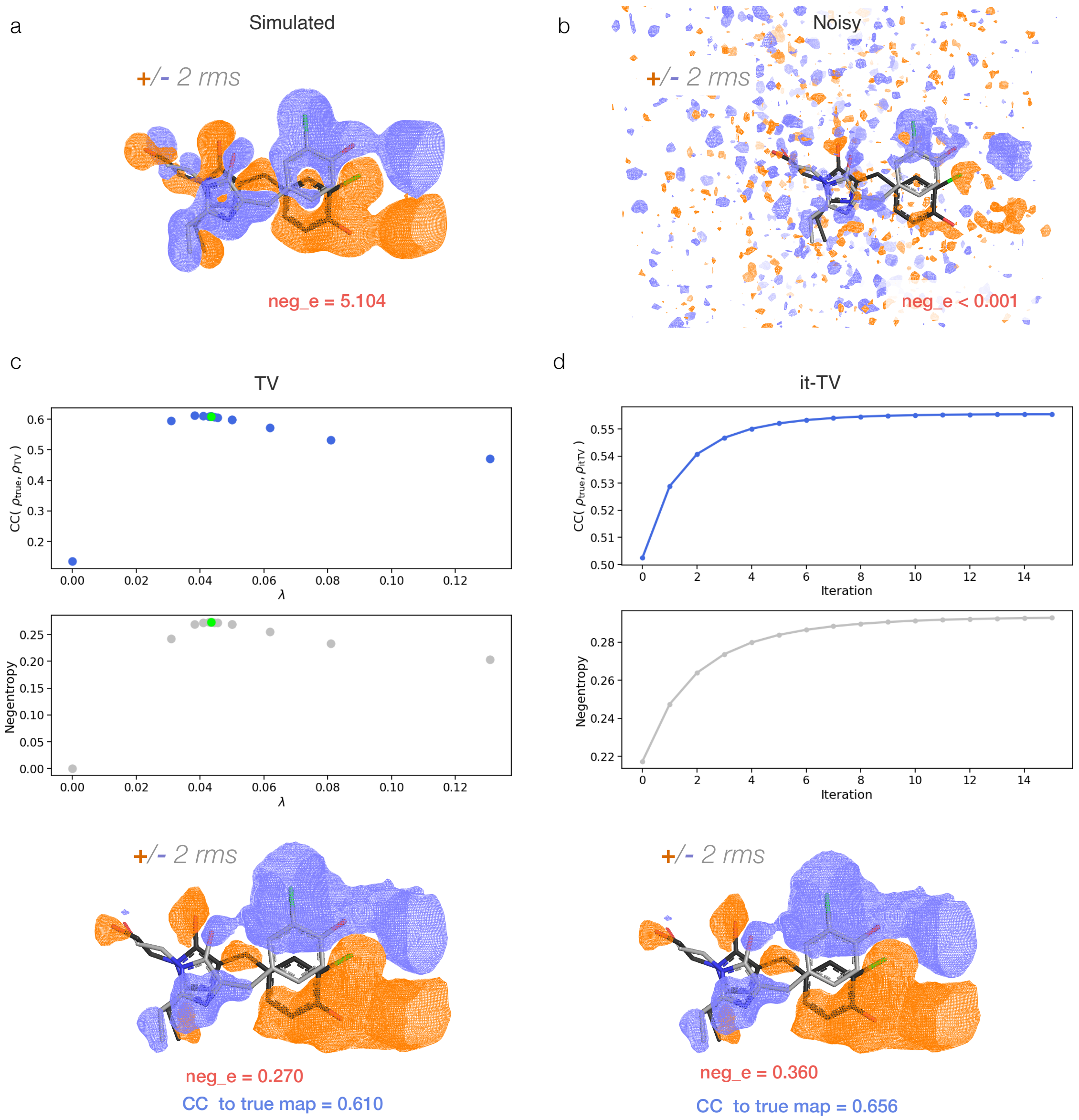

Figure S3. Validation of total variation denoising with a synthetic noisy map. (a) Simulated *trans*-to-*cis* difference density map. **(b)** Noise introduced by adding random values to the *trans* and *cis* structure factors, sampled from a Gaussian distribution with mean 0 and standard deviation equal to 10% the standard deviation of the structure factor magnitudes. The real-space map thus displays both the signal and the added noise. **(c)** Negentropy values for the denoised map across a range of regularization weights. The peak negentropy closely matches the λ value that maximizes the real-space Pearson correlation coefficient between the denoised map and the true calculated map (ρ_{true}). **(d)** Application of the iterative-TV (it-TV) technique further refines the denoised map and results in improved negentropy and correlation to ground truth map values. As iterations progress, the denoised map (ρ_{itTV}) increasingly approximates the ground truth map (ρ_{true}), with negentropy plateauing to a maximum, supporting its use as a robust quality metric.

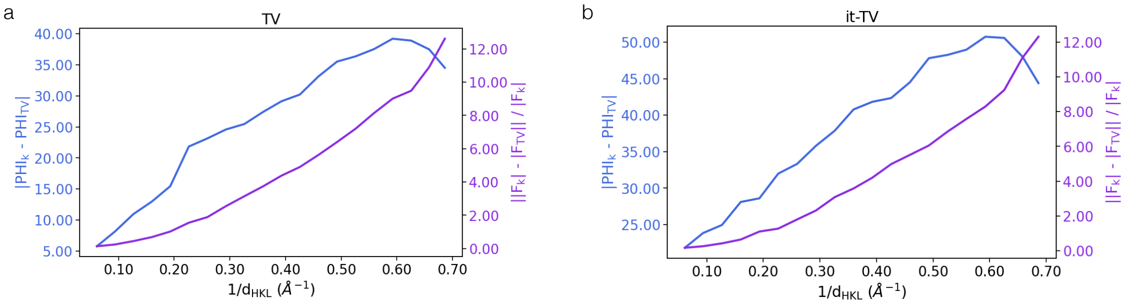

Figure S4. TV denoising primarily modifies high resolution reflections. Structure factor amplitudes and phase differences between the k-weighted map for the PDB ID 8A6G Cl-rsEGFP2 test case and the map generated from **(a)** single pass TV denoising and **(b)** iterative-TV. The average statistics are plotted after binning the datasets in 20 resolution shells.

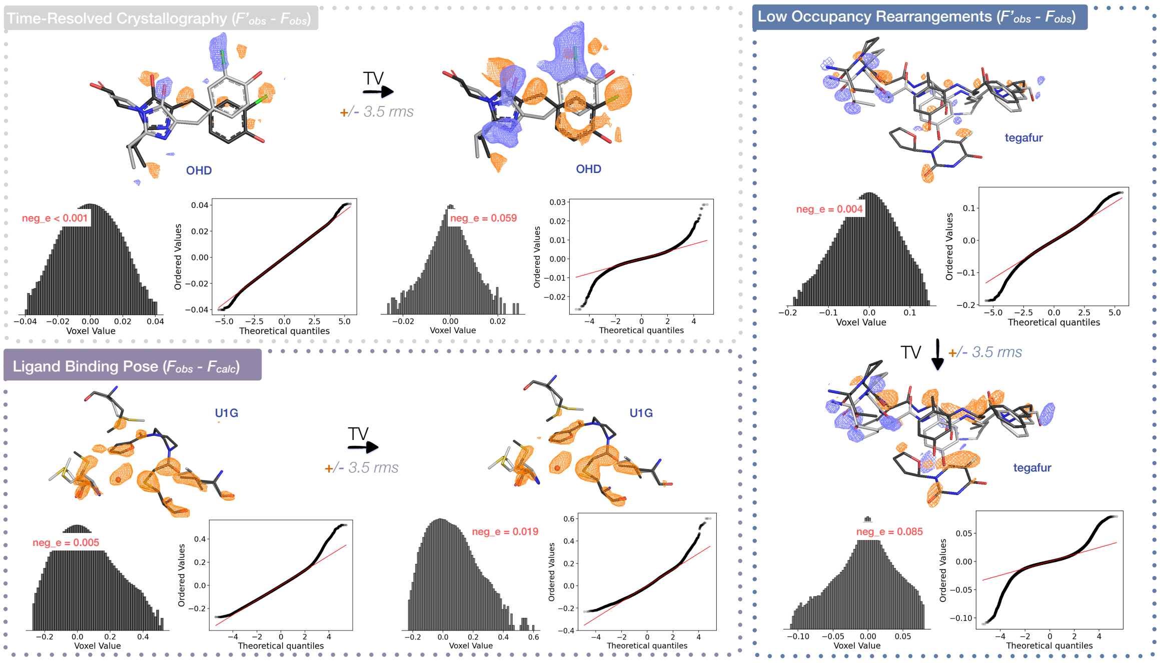

Figure S5. Map denoising through TV minimization is applicable to a range of science cases. The best negentropy DED map after a single pass of TV denoising at its refined λ is displayed for the three crystallographic case studies outlined in the text. Reference state structures are shown in gray, while structures that were refined to the perturbed dataset are shown in black. Voxel value histograms for the maps (with a log-scale on the y-axis), probability plots, and respective negentropy values (neg_e) are also reported.

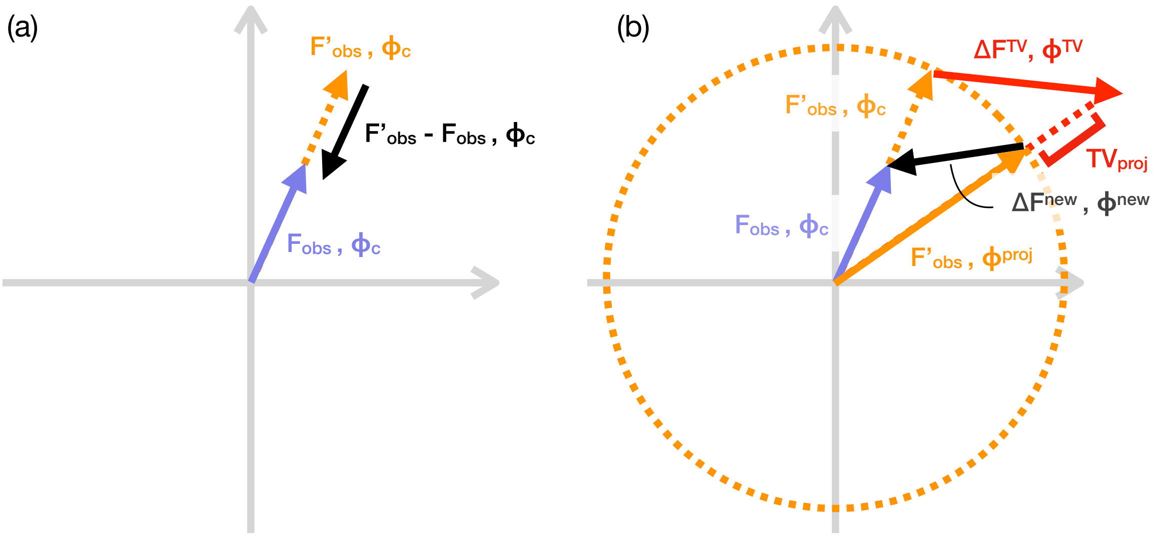

Figure S6. TV-denoised structure factors can be used to better estimate the phases of an F' state. (a) Standard isomorphous Fourier difference syntheses are based on the approximation that the native **F** and the derivative **F'** share a phase calculated from a reference state (ϕ_c). This approximation introduces a source of error, which is expected to halve the signal-to-noise ratio in the final map [5] **(b)** Our iterative method proposes an improvement: we first apply TV denoising to the initial difference map, then perform an inverse Fourier transform to obtain complex difference structure factors, $\Delta\mathbf{F}^{\mathbf{TV}}$. We then project each vector onto a circle centered at the origin with radius $|F'_{obs}|$, producing an updated estimate of the derivative phases (ϕ^{proj}). These phases are used to generate a new difference map (ΔF^{new}, ϕ^{new}), which is denoised again, and the cycle is repeated until convergence.

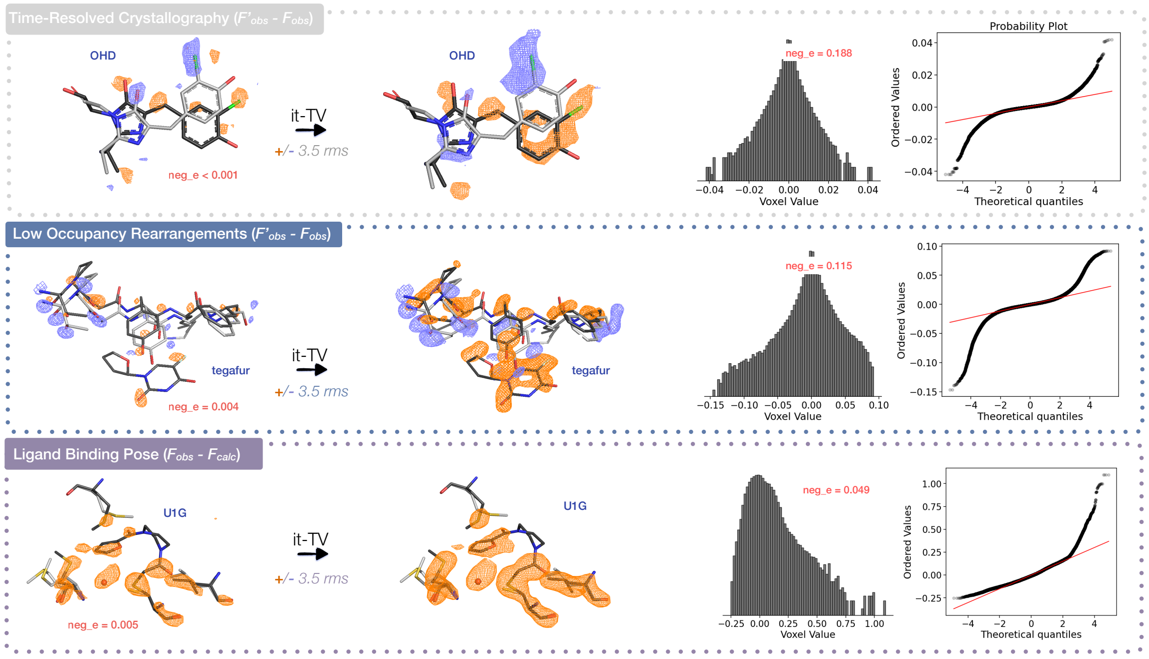

Figure S7. An iterative TV minimization algorithm estimates the phases for low occupancy states. We show it-TV maps for our three test datasets, with their voxel value histogram, probability plots, and associated negentropy. Reference state structures are shown in gray, while structures that were refined to the perturbed dataset are shown in black.

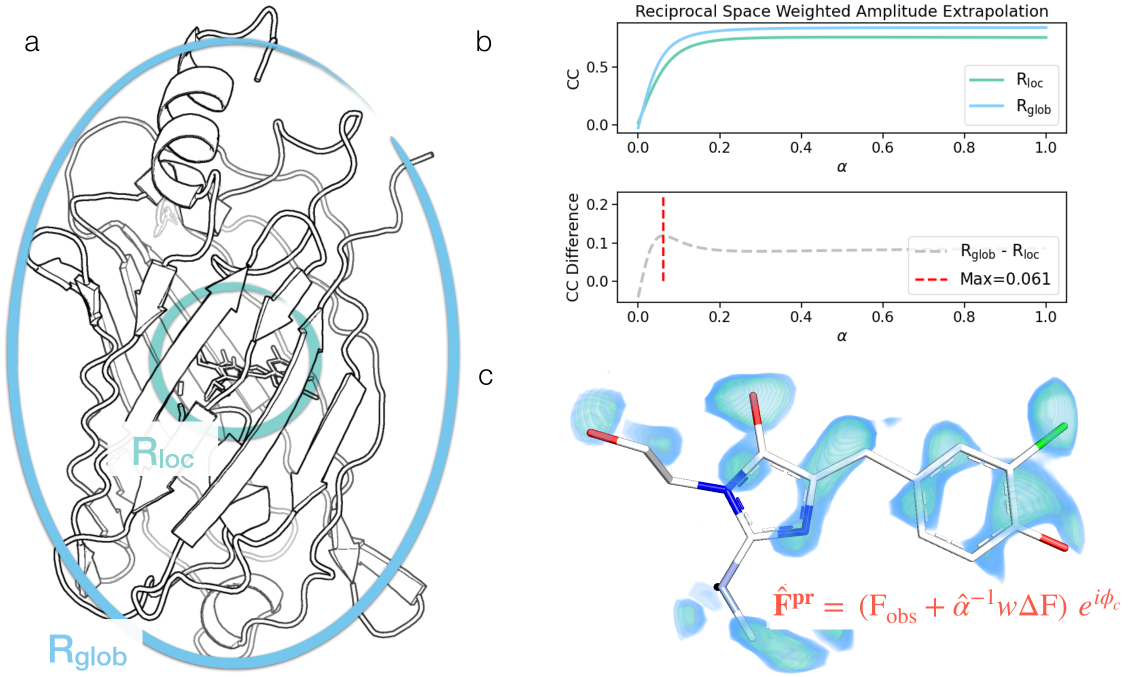

Figure S8. Reciprocal space background subtraction for extrapolating the density of perturbed states. (a) To find an estimate for the extrapolation factor α, we re-implement the background correction carried out by the PanDDA suite [6]: in real space, we define a local region, R_{loc}, based on where the strongest signals in the METEOR it-TV map are found (for the Cl-rsEGFP2 chromophore pocket shown here, R_{loc} is set as a 5Å sphere centered on the chromophore isomerizing bond). The entire protein is defined as R_{glob} after a solvent mask is applied. Fourier amplitudes of the form: $F^{pr} = (F_{obs} + \alpha^{-1} w\Delta F)$ are computed for a range $0 \leq \alpha \leq 1$. The phases from the reference model (ϕ_c) are used for map generation. **(b)** For each value of α, the Pearson correlation coefficient between the respective $\mathbf{F^{pr}}$ map and the map obtained from the reference model calculated structure factors (F_c) is computed. This is done for both R_{loc} and R_{glob}. The fraction of α that maximizes the difference between these two correlation coefficients ($\hat{\alpha}$) is chosen to approximate the structure factors of the perturbed state $\mathbf{F^{pr}}$. **(c)** The extrapolated map obtained for the picosecond Cl-rsEGFP2 photoisomerization data is shown: the outline of the *cis* photoproduct species becomes clear and can confidently support the presence of the new species.

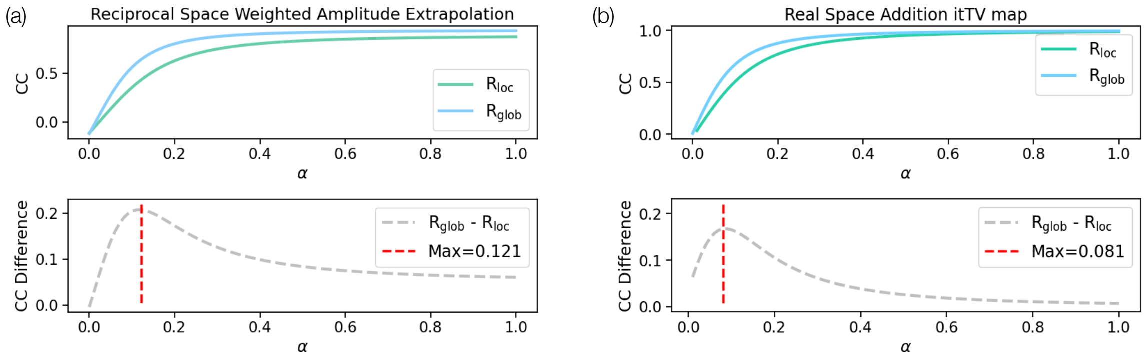

Figure S9. Estimate of α parameter for map extrapolation for the M^{pro}-tegafur complex. Determination of the α parameter for map extrapolation is carried out with our reciprocal space **(a)** and real space **(b)** implementations as described in the Methods section. For both cases, the local sphere is centered on the ligand and chosen with a radius of 8 Å. Note that, when the it-TV map is used for the extrapolation, the expectation that α should be half of the perturbed state occupancy no longer holds (as we are no longer approximating **F** and **F'** using the same phase).

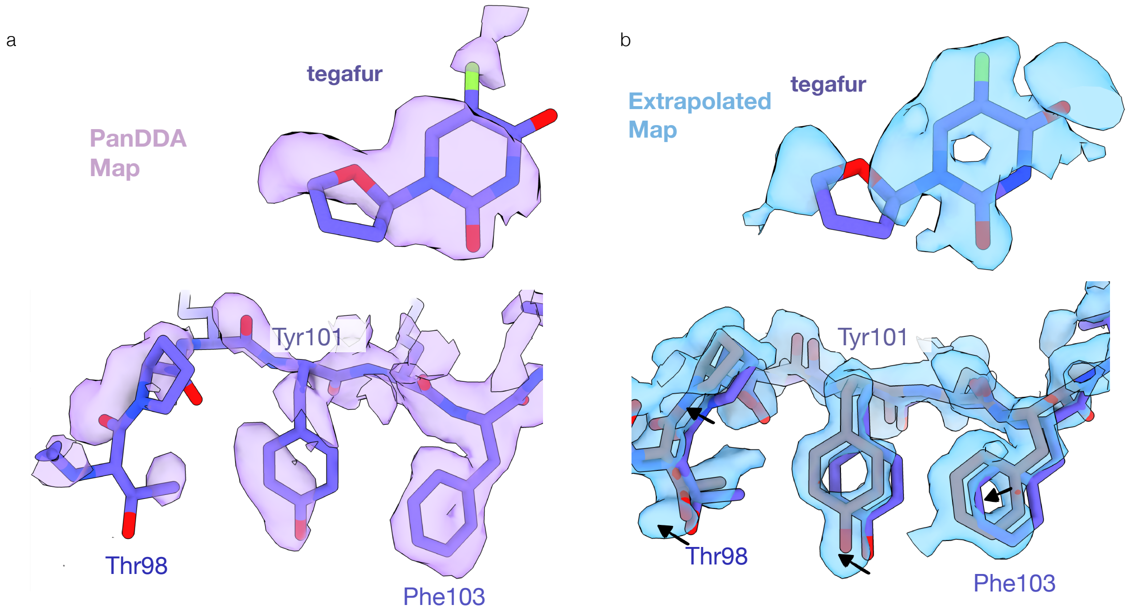

Figure S10. Extrapolated density for the M^{pro}-tegafur complex obtained using reciprocal space background subtraction The PanDDA map **(a)** for the M^{pro}-tegafur dataset (PDB ID 7AWR) is compared to the $\hat{F}^{pr}$ map **(b)** obtained from the reciprocal space map extrapolation procedure outlined in Figures S8 and S9(a). The extrapolated $\hat{F}^{pr}$ map displays clear outlines for Thr98, Tyr101, and Phe103 as well as backbone density that can be interpreted as rearrangements away from the fragment binding pocket and that can be used for refinement of new atomic positions. The resolution in the $\hat{F}^{pr}$ map is improved compared to the PanDDA map: ring structures and oxygen densities are considerably more interpretable for both the fragment density and the protein side chains.

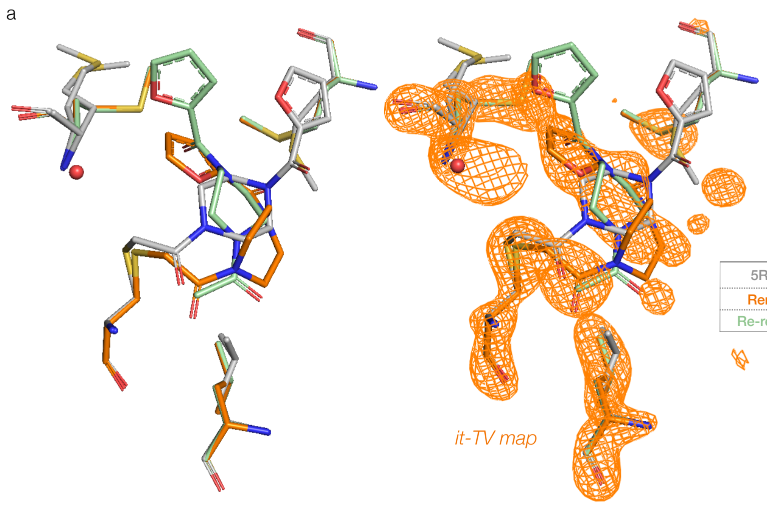

b

	Ligand RSCC	R-work	R-free
5RGO – deposited	0.513	0.1952	0.234
Remodeled to it-TV	**0.630**	**0.1716**	**0.230**
Re-refined with phenix	0.487	0.1717	0.227

Figure S11. Ligand binding pose modeling to iterative-TV density. (a) The deposited structure for the M^{pro}-U1G complex (PDB ID 5RGO) is shown in gray. The structure that was manually remodeled to the density from the iterative-TV map derived in Figure 4 of the main text is shown in orange. The model that can be obtained by re-refining the deposited model with phenix.refine [7] is shown in green. The three structures are displayed with (right) and without (left) overlaying the it-TV map. **(b)** Real-space correlation coefficient (RSCC) values for ligand density between the model calculated map and the ligand polder map [8] (calculated from the original model and the deposited structure factors) are reported in the first column. R-factor values for the newly refined models and the original deposition are also reported. The structure that was remodeled to the it-TV map displays improved fit to the data as assessed by RSCC and R-factors.

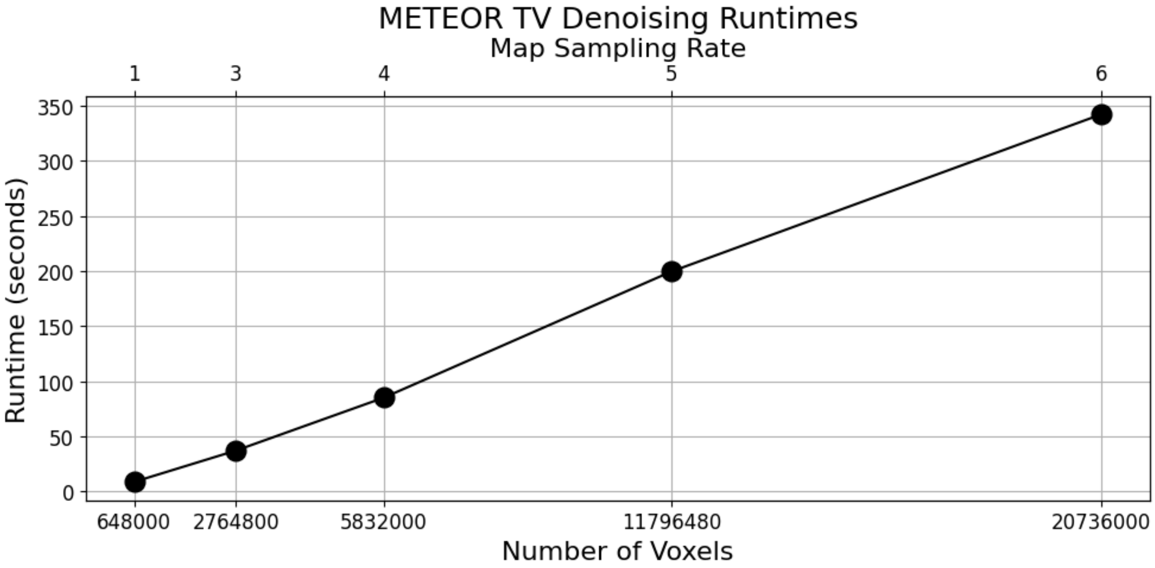

Figure S12. Computation Times for a Full TV Denoising Protocol with METEOR. Run times for the full single-pass TV denoising protocol of the P212121 Cl-rsEGFP2 dataset (unit cell dimensions: 51.99 62.91 72.03 90.00 90.00 90.00, resolution: 1.6 Å). A map sampling rate of 4, as defined by GEMMI/CCP4 is standard.

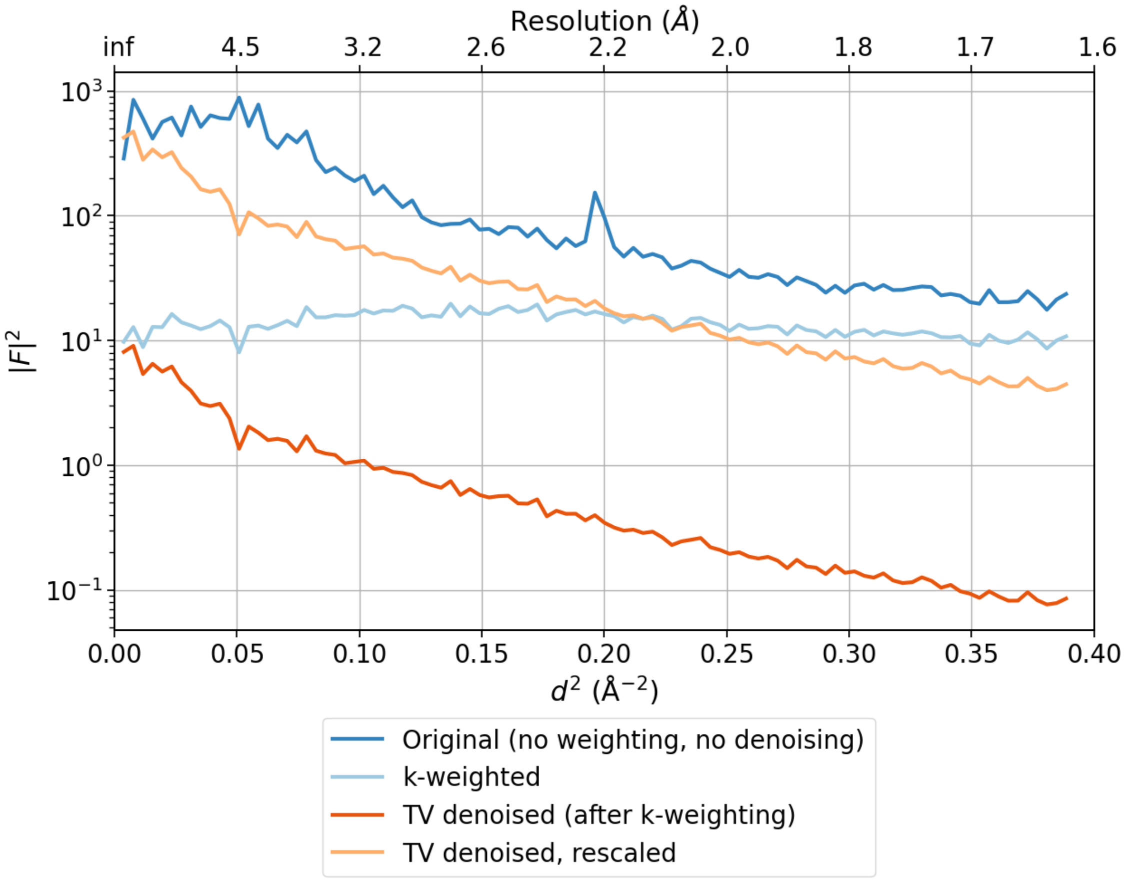

Figure S13. Common Scale for DED Maps. Schemes that modify difference structure factor amplitudes affect the total power of the related DED map. We show how this is true for the Cl-rsEGFP2 data used throughout the main text ("original" refers to a vanilla Fo-Fo map). Both k-weighting and subsequent TV denoising result in a reduction in power compared to a simple Fo-Fo map. To ensure that METEOR outputs maps on the same scale as the original inputs, we rescale the weighted or denoised map back to its corresponding original difference map using data that lie below 1.5 Å of the stated resolution cutoff.

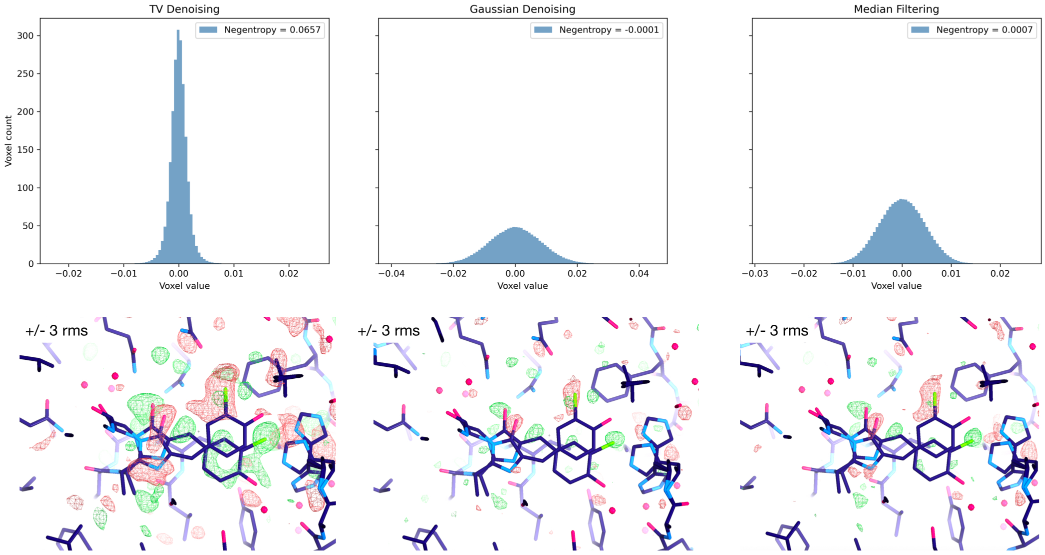

Figure S14. Comparison of Different Denoising Filters. We report the performance of TV denoising to Gaussian and median filtering on our benchmark Cl-rsEGFP2 DED map. The TV-denoised map displays visually stronger and more interpretable chromophore signals. This qualitative impression was supported by higher negentropy values, which we use as a proxy for signal content.

References

1. Rudin, L.I.; Osher, S.; Fatemi, E. Nonlinear total variation based noise removal algorithms. *Physica D: Nonlinear Phenomena* **1992**, *60*, 259–268. doi:10.1016/0167-2789(92)90242-F.
2. Chambolle, A. An Algorithm for Total Variation Minimization and Applications. *Journal of Mathematical Imaging and Vision* **2004**, *20*, 89–97. doi:10.1023/B:JMIV.0000011325.36760.1e.
3. Ren, Z.; Perman, B.; Šrajer, V.; Teng, T.Y.; Pradervand, C.; Bourgeois, D.; Schotte, F.; Ursby, T.; Kort, R.; Wulff, M.; Moffat, K. A Molecular Movie at 1.8 Å Resolution Displays the Photocycle of Photoactive Yellow Protein, a Eubacterial Blue-Light Receptor, from Nanoseconds to Seconds †. *Biochemistry* **2001**, *40*, 13788–13801. doi:10.1021/bi0107142.
4. De Zitter, E.; Coquelle, N.; Oeser, P.; Barends, T.R.M.; Colletier, J.P. Xtrapol8 enables automatic elucidation of low-occupancy intermediate-states in crystallographic studies. *Communications Biology* **2022**, *5*, 640. doi:10.1038/s42003-022-03575-7.
5. Henderson, R.; Moffat, J.K. The difference Fourier technique in protein crystallography: errors and their treatment. *Acta Crystallographica Section B Structural Crystallography and Crystal Chemistry* **1971**, *27*, 1414–1420. doi:10.1107/S0567740871004060.
6. Pearce, N.M.; Krojer, T.; Bradley, A.R.; Collins, P.; Nowak, R.P.; Talon, R.; Marsden, B.D.; Kelm, S.; Shi, J.; Deane, C.M.; von Delft, F. A multi-crystal method for extracting obscured crystallographic states from conventionally uninterpretable electron density. *Nature Communications* **2017**, *8*, 15123. doi:10.1038/ncomms15123.
7. Adams, P.D.; Afonine, P.V.; Bunkóczi, G.; Chen, V.B.; Davis, I.W.; Echols, N.; Headd, J.J.; Hung, L.W.; Kapral, G.J.; Grosse-Kunstleve, R.W.; McCoy, A.J.; Moriarty, N.W.; Oeffner, R.; Read, R.J.; Richardson, D.C.; Richardson, J.S.; Terwilliger, T.C.; Zwart, P.H.; IUCr. PHENIX: a comprehensive Python-based system for macromolecular structure solution. *Acta Crystallographica Section D Biological Crystallography* **2010**, *66*, 213–221. doi:10.1107/S0907444909052925.
8. Liebschner, D.; Afonine, P.V.; Moriarty, N.W.; Poon, B.K.; Sobolev, O.V.; Terwilliger, T.C.; Adams, P.D. Polder maps: improving OMIT maps by excluding bulk solvent. *Acta crystallographica. Section D, Structural biology* **2017**, *73*, 148–157. doi:10.1107/S2059798316018210.
