# Supplementary material for: Denoising and iterative phase recovery reveal low-occupancy populations in protein crystals
Source: Commun Biol. 2025 Nov 24;8:1649. doi: 10.1038/s42003-025-09031-6 (PMC12644873; doi:10.1038/s42003-025-09031-6)
Supplement: Supplementary file 3 — Description of Additional Supplementary files [file 42003_2025_9031_MOESM3_ESM.pdf]

Description of Additional Supplementary Files

File name: Supplementary Data 1
Description: Data for Figure 2 power spectra

File name: Supplementary Data 2
Description: Data for Figure 2 negentropy screen

File name: Supplementary Data 3
Description: Data for Figure 3 graph

File name: Supplementary Data 4
Description: Data for Figure 5 graph
